# The Association between Family and Parental Factors and Obesity among Children in Nanchang, China

**DOI:** 10.3389/fpubh.2016.00162

**Published:** 2016-08-12

**Authors:** Peng Zhang, Hongjiao Wu, Xiaojun Zhou, Yuanan Lu, Zhaokang Yuan, Justin B. Moore, Jay E. Maddock

**Affiliations:** ^1^School of Public Health, Nanchang University, Nanchang, Jiangxi, China; ^2^Office of Public Health Studies, University of Hawaii at Manoa, Honolulu, HI, USA; ^3^Department of Health Promotion, Education, and Behavior, Arnold School of Public Health, University of South Carolina, Columbia, SC, USA; ^4^Department of Environmental and Occupational Health, School of Public Health, Texas A&M University, College Station, TX, USA

**Keywords:** obesity, China, family factors

## Abstract

**Background:**

With rapid economic development in China, traditional patterns of health behaviors are changing, concurrent with a rise in childhood obesity. While the home environment and parenting behaviors are modifiable factors that could be targeted for intervention, little is known about their relationship with children’s health behaviors. Therefore, the purpose of this study was to investigate the relationship between obesity and home and parenting factors in Chinese children.

**Methods:**

A cross-sectional survey was conducted in Nanchang, China in 2013 with caregivers (*N* = 470) of a child between the ages of 2 and 10 years. Regression analyses were conducted to determine risk factors for childhood obesity.

**Results:**

Obesity prevalence (21.7%) did not differ by demographic variables. Eight physical activity, nutrition, and sedentary variables had significant relationships to obesity status. Logistic regression analysis revealed three significant predictors of obesity: the number of days the family eats meals together (odds ratio = 0.84, 95% CI 0.73–0.96) and parental home computer use time (odds ratio = 0.83, 95% CI 0.72–0.96) were related to lower levels of obesity, while parental television time (odds ratio = 1.25 95% CI 1.07–1.47) was related to higher levels of obesity.

**Conclusion:**

The prevalence of obesity among children is high in Nanchang. Family and environmental risk factors are significantly related to obesity.

## Introduction

Overweight and obesity are becoming more prevalent worldwide, even among children in developing countries (1). Since obesity during childhood greatly increases the likelihood of adult obesity (2–4), the prevention of childhood obesity is becoming an important global public health issue, as obesity is important risk factor in cardiovascular, cerebrovascular, metabolic, and other non-communicable diseases (5). A study from the United States showed that almost 40% of the children who were overweight or obese in childhood would be classified as obese when they were 30 years old compared with only 5% of normal weight children (6). Childhood obesity is also significantly linked to morbidity and mortality in adulthood (7).

With the rapid economic development of China in the recent years, traditional physical activity, sedentary, and nutrition patterns are becoming more westernized. In China, from 1993 to 2009, obesity increased from about 3 to 11% in men and from 5 to 10% in women (8). A recent representative study of youth in China has also shown a dramatic rise in obesity. In 1985, the prevalence of overweight or obesity in urban boys, urban girls, rural boys, and rural girls was 1.3, 1.6, 0.5, and 1.6%, respectively. By 2010, the prevalence of overweight or obesity in these groups increased to 23.2, 12.7, 13.8, and 8.6%, respectively (9). This is significant, since obese children in China are more likely to have hypertension than non-obese children (10). Unlike many Western countries, childhood obesity rates are highest in families with higher socioeconomic status (11, 12); therefore, research conducted in western countries cannot be generalized to Chinese citizens.

Childhood obesity is caused by diverse and interrelated factors, including genetics, birth weight, diet, exercise, parental behavior awareness, socioeconomic, and psychological factors, among others (13). Recent studies in China have shown similar correlates of obesity as observed in Western countries, which includes not eating breakfast, soft drink intake, frequent intake of high-energy, but low-nutrition sweets and snacks and having dinner while watching television and eating before bedtime (14, 15). Social cognitive theory proposes that observational learning, modeling, and environmental factors are important predictors of behavior (16). As such, parents’ behaviors can directly affect to the development of lifestyle habits in children (17). In China, the majority of urban families only have one child in accordance with the Chinese government’s one child policy. As a result, parents and grandparents often have active roles in the child’s feeding and lifestyle activities. A study in Beijing revealed that grandparents’ nutritional education is correlated with children developing good eating behaviors if the three generations live together (18). A review of the effects of parental attitudes toward children’s eating behavior in western countries has shown a strong effect. Two factors, restriction of certain foods and pressure to eat healthy foods along with the family food environment, have shown to be related to childhood overweight and obesity (19). However, little research has been conducted to assess the possible effect of the home environment and parental practices on childhood obesity in China.

Nanchang is the capital of Jiangxi province, which is one of the poorest provinces in China. With a population over 5 million in 2010, Nanchang has a rapidly developing economy, with a Gross Domestic Product exceeding 300 billion RMB in 2012, a 12.5% increase over the previous year. However per capita GDP is only 58,715 RMB (approximately USD $9,625) (20). The problem of childhood obesity has been well documented in high- and middle-income countries but seen less often in low-income countries. Little is known about the development of childhood obesity as a community transitions from low- to middle-income areas. As such, Nanchang represents an ideal setting to study factors that influence childhood obesity due to the transitioning nature of the economy and the lifestyle of the residents. Since the rapidly growing childhood obesity rates in China’s urban areas are a major public health concern, it is imperative to identify potentially modifiable correlates that can be targeted for intervention. However, while studies in developed countries have shown a strong relationship between home and parental factors and childhood obesity, little is known about these relationships in China (21). Therefore, the purpose of this study was to investigate the relationship between childhood obesity, parenting behaviors, and the home environment in a sample of children in urban Nanchang. Results of this study can be used to focus interventions to help control the rise of obesity in China.

## Materials and Methods

### Procedures

A cross-sectional survey was collected between May and June 2013 at three sites in Nanchang, Jiangxi, and China. Sites included two vaccination clinics run by the China Centers for Disease Control and a children’s hospital. Members of the investigating team had existing relationships with the three sites, which serve the majority of the Nanchang population who live in two of the nine major districts of the city. Because this was an exploratory study, we were unable to calculate power *a priori*. We aimed to collect at least 400 completed surveys to have adequate numbers of both genders and overweight and obese children. The Institutional Review Boards of the University of Hawaii at Manoa and Nanchang University approved the study.

### Inclusion Criteria

Nanchang residents aged 18 years or older who were caregivers of a child between the ages of 2 and 10 years old were eligible. Interviewers underwent an extensive training session on survey data collection methodology to ensure rigorous adherence to interview protocols. Interviewers approached every possible participant who had a child appearing to be older than 2 years. Those who qualified were asked if they were willing to participate. If they consented, the face-to-face survey was administered.

### Measures

A modified version of the Healthy Homes Survey was used as the main study instrument (21). This survey was developed in the United States and has good reliability and validity. The main modification to the survey was to substitute food items found in the American home with a list of the most common foods reported in Chinese households from the Shanghai Women’s Health Study (22). This list of food items has been previously used in China and has been shown to be valid and reliable (22). Items were translated and back translated by the research teams in China and the United States. Minor wording changes were included to make the questions clear and understandable in Mandarin. A 12-part questionnaire, which included 146 items that focused on home environment, comprised the survey. Each survey took on average 15–25 min to complete through face-to-face interviews. Surveys were extensively pre-tested with Nanchang caregivers prior to launching the study to ensure comprehension and face validity in the local population.

For this paper, data from four main sections were selected. These included: demographics, physical activity, sedentary behavior, and nutrition-related variables. Demographic variables included the respondent’s age, sex, height, weight, household income, food insecurity, and type of residence, along with the child’s general health status, age, sex, height, and weight. Food insecurity was assessed on a four-point scale by how often the family did not have enough to eat. Physical activity support was indicated as the sum of the presence of nine common sports related items in the home. A 6-item scale (α = 0.77) assessed participants’ encouragement of their child’s physical activity. The child’s participation in organized sports and the adult’s attendance at a gym or exercise classes was assessed. Variables related to sedentary behavior included having a television in the child’s room; child’s ownership of a handheld gaming device or video game system, time spent watching television or playing video games, and a 3-item scale on parental rules restricting the use of television and video games (α = 0.89). Fruit availability was operationalized as an index of how often eight common fruits were available in the household, and the vegetable availability consisted of how often sixteen common vegetables were available in the household. The availability of sugared sodas, diet sodas, other sugar-sweetened beverages, and fruit juice was also assessed. All availability quests were asked on a 5-point scale from 0 = never to 4 = always. Several eating habit questions were also included in this survey study, including how often the family eats dinner together (# of days per week, 0–7), how often the child eats breakfast, how often dinner includes freshly prepared foods, how often dinner included processed foods, and how often sodas or other sweetened beverages were served at meals and snacks (response options 0 = almost never to 3 = almost always). Parental food rules consisted of 8 items measuring the strength of household regulations and restrictions on the child’s eating behavior (α = 0.70). Parental behavior, including how often parents’ watch television, use computers at home, play video games, and play active video games, was also assessed (response options measured as time per day; 0–4 h a day).

### Child’s Weight Status

The children’s height and weight were collected by self-report from the caregivers. A subset of participants’ height and weight were validated by objective measures collected at the CDC sites. Body mass index (BMI) was calculated using the height and weight reported on the survey. The CDC BMI growth chart was used to determine age- and sex-specific BMI percentiles (BMI%) used to categorize children as underweight (BMI% < 5th percentile), normal weight (5th ≤ BMI% < 85th), overweight (85th ≤ BMI% < 95th), or obese (BMI% ≥ 95th) (23).

### Data Analysis

The prevalence of obesity was calculated, stratified by age, and sex. Descriptive statistics were calculated for the children’s demographic characteristics, physical activity, sedentary behaviors, and eating habits. Bivariate analyses (*t*-tests and chi-squares) were used to assess the relationship between obesity and potential risk factors. Logistic regression models with obesity as the dependent variable (obese = 1, not obese = 0) were then run with all variables that had a *p*-Value less than.10 in the bivariate analyses. Significance was set at *p* < 0.05 for all analyses, which were conducted using SPSS 20.0 (IBM, New York, NY, USA).

## Results

### Sample Characteristics

In total, 480 surveys were completed (response rate = 87.9%), and 470 surveys with complete height and weight data were retained for analysis. More than half (61.1%) of the respondents were the child’s mothers. Slightly more than half of the children (54.7%) were boys. About half (58.7%) of caregivers believed their child’s health was excellent or very good. A minority (38.7%) of families’ household income was higher than 60,000 RMB (approximately USD $10,000) per year. Most caregivers (64.0%) worked full time outside the home, and most families (87.4%) lived in an apartment or condominium.

### Prevalence of Obesity by Demographics

About one in five (21.7%) of the children had BMI scores in the obese range. Obesity was more prevalent in boys (24.9%) than in girls (17.8%), but this difference was not statistically significant (*p* = 0.064). Obesity prevalence did not differ by either the caretaker’s age or the child’s age. Children from low-income households (26.2%; less than ¥30,000 annually) were more likely to be obese than middle (18.0% for ¥30,000–60,000) and high-income households (19.7% for more than ¥60,000). Obese children were also more likely to come from households with higher levels of food insecurity. General health status was not related to obesity. Demographics by children’s obesity status are presented in Table 1.

**Table 1:** **Demographic characteristics by children’s obesity classification**.

Variable	Non-obese (*n* = 368)	Obese (*n* = 102)	*p*-Value*
Children’s age in years, mean (SD)	4.7 (2.6)	4.8 (2.3)	n.s.
Caregivers’ age in years, mean (SD)	36.1 (10.2)	38.2 (11.7)	n.s.
**Child’s sex**			
% Male	52.4%	62.7%	n.s.
**Health status**			
Excellent or very good *“n* = 276”	57.6%	62.7%	n.s.
**Household income in RMB per year**			
Less than 30,000 “*n* = 139”	73.8%	26.2%	n.s.
30,000–60,000 *“n* = 149”	82.0%	18.0%	
More than 60,000 “*n* = 182”	80.3%	19.7%	
**Food insecurity**			
Sometimes we do not have enough to eat	0.0%	2.9%	<0.01
We have enough to eat but not always the kind we want	18.8%	22.5%	
We always have enough to eat and the kind we want	81.2%	74.5%	

*Chi-square test was used for categorical variables; t-test for continuous variables.

### Physical Activity Supports and Sedentary Risk Factors

Several supportive and risk factors were examined to see if they were related to obesity in the children. Non-obese children (*M* = 4.7) had significantly more physical activity supports (e.g., bicycle and jump rope) than obese children (*M* = 4.1, *p* < 0.05). There was no significant difference in obesity prevalence between children participating in organized sports and children who did not. Participants’ encouragement of their child’s physical activity differed significantly by weight status, with caregivers of obese children providing less encouragement (*M* = 20.8, SD = 3.8) than parents with non-obese children (*M* = 21.6, SD = 3.5, *p* < 0.05). However, caretakers of non-obese children did not report going to the gym or exercise classes more often than parents of obese children. Non-obese children (*M* = 4.05, SD = 1.87) had more parental restrictions on use of television and video games than obese children (*M* = 3.51, SD = 1.84, *p* < 0.05).

Analyses showed no significant differences in the prevalence of obesity by presence of a television in the child’s room or ownership of a handheld gaming device. Non-obese children (42.1%) were more likely to have a video game station or computer than obese children (28.4%, *p* < 0.05). Table 2 displays physical activity supports and sedentary risk factors by obesity status.

**Table 2:** **Physical activity and sedentary behaviors by child’s obesity classification**.

Variable	Non-obese (*n* = 368)	Obese (*n* = 102)	*p*-Value*
Availability of sports/physical activity equipment in the house (0–9)	4.67 (1.93)	4.15 (2.19)	<0.05
Participation in organized sports or exercise classes (% yes)	54.3%	50.0%	n.s.
Child’s participation in physical activity during free time (% often/always)	58.4%	62.7%	n.s.
Parent’s encouragement of exercise	21.6 (3.5)	20.8 (3.8)	<0.05
Parent’s attendance at gyms and exercise classes	2.82 (2.02)	2.89 (2.07)	n.s.
Does your child have a TV in their bedroom? (% yes)	26.9%	30.4%	n.s.
Does your child have a video game station or computer?	42.1%	28.4%	<0.05
Does your child have a handheld gaming device?	27.7%	22.5%	n.s.
Parental rules regarding use of TV and video games (0–7)	4.05 (1.87)	3.51 (1.84)	<0.05

*Chi-square test was used for categorical variables; t-test for continuous variables.

### Nutritional Risk Factors

Nutritional variables were then examined for their possible association with obesity status. Some variables showed little/no variability and were removed prior to data analyses. This included the child eating breakfast (97.4% often/always), families eating fruits and vegetables with their main meal (87.9% often/always), and the family freshly preparing food for the main meal (100% often/always). No significant differences by obesity status were seen for availability of fruits/vegetables, sugary soft drinks, diet sodas, or fruit juice in the home. Rules around food consumption did not differ by child’s obesity status. Table 3 displays the nutrition variables by obesity status.

**Table 3:** **Nutrition-related variables by child’s obesity classification**.

Variable	Normal weight (*n* = 405)	Overweight/obese (*n* = 108)	*p*-Value*
**Household availability of**			
Vegetables	55.2 (8.4)	54.8 (8.1)	n.s.
Fruit	27.6 (4.2)	26.8 (4.6)	n.s.
Sugary soft drinks	2.02 (0.92)	1.94 (0.85)	n.s.
Other sugary drinks	2.46 (1.07)	2.41 (0.99)	n.s.
Diet soft drinks	1.93 (1.03)	2.01 (1.07)	n.s.
Fruit juice	6.97 (3.12)	6.92 (3.34)	n.s.
**How often do you buy the following at your child’s request?**			
Fruits and vegetables	3.93 (0.78)	3.88 (0.72)	n.s.
Snacks or sugary cereal	2.95 (1.05)	2.97 (1.08)	n.s.
**Rules regarding food consumption**	12.77 (2.18)	13.00 (2.07)	n.s.
**How many days of the week does your family eat dinner together?**	6.51 (1.38)	6.11 (1.93)	<0.05

*Chi-square test was used for categorical variables; t-test for continuous variables.

### Parental Modeling

Parents with obese children watched more television (*M* = 4.09 SD = 1.42 vs. *M* = 3.70, SD = 1.5, *p* < 0.05) but spent less time working on the computer (*M* = 2.82, SD = 1.75 vs. 3.38 SD = 1.71, *p* < 0.05) than parents of non-obese children. The time parents spent playing video games or active video games was not significantly associated with their child’s weight status.

### Logistic Regression

The eight variables in the bivariate analysis that had *p*-Values <0.10 were entered into a logistic regression with the child’s obesity status as the dependent variable. The overall regression was significant [χ^2^(8) = 32.7, *p* < 0.001], and three variables were found to be significant (*p* < 0.05). Protective factors included the number of days the family eats meals together (odds ratio = 0.84, 95% CI 0.73–0.96) and parental home computer use time (odds ratio = 0.83, 95% CI 0.72–0.96). Parental television time (odds ratio = 1.25 95% CI 1.07–1.47) was related to higher levels of obesity. Table 4 displays the results of the logistic regression.

**Table 4:** **Logistic regression by obesity (*n* = 469)**.

Overall: χ^2^(8) = 31.7, *p* < 0.001			
Variable	Odds ratio	CI 95%	*p*-Value
Food insecurity	1.42	0.82–2.48	n.s.
Availability of sports/physical activity equipment in the house	0.99	0.87–1.13	n.s.
Parent’s encouragement of exercise	0.99	0.93–1.06	n.s.
Does your child have a video game station or computer?	0.69	0.41–1.16	n.s.
Parental rules regarding use of TV and video games?	0.90	0.79–1.02	=0.116
How many days of the week does your family eat dinner together?	0.84	0.73–0.96	<0.05
Parent’s computer time	0.83	0.72–0.96	<0.05
Parent’s television time	1.25	1.07–1.47	<0.05

## Discussion

Obesity is becoming a major public health problem in China similar to Western countries. The prevalence of obesity in the present sample was 21.7%. This rate is similar to the combined overweight and obesity prevalence of 17.8 and 21.7% found in Chengdu (2011) and Beijing (2004) with the same BMI reference points (24, 25). The prevalence of obesity in China from our survey is similar to obesity rates reported in the United States (26, 27). This rapid change of obesity status is striking when compared to a sample from 1985, where childhood obesity was almost non-existent in China (9).

In this study, we examined home and parental factors among obese children compared to non-obese children. While obesity rates have been rising rapidly in the country, several findings from this study are of interest and encouraging. Families still typically eat dinner together every night, and fresh food is always prepared. The number of nights a family eats together provides a protective factor for obesity with about a 16% reduction for every extra night the family eats together. However, similar to the United States, almost every house has a television set and video gaming systems were popular among the youth. Access to physical activity equipment did protect somewhat against obesity. Unlike other studies, this study did not show a relationship between children television viewing and obesity (28–30). However, parental rules toward media use were related to childhood obesity. This may indicate a shift in the importance of traditional television viewing toward overall screen time, which can be more difficult to monitor especially given the proliferation of handheld gaming devices, tablet devices, and smart phones. Parental television use and computer use was related to obesity with every hour of increased television time related to a 25% increase in obesity. Parental computer use time was a protective factor. It is unclear exactly why this would be true and may be related to socioeconomic status. Social cognitive theory addresses the importance of modeling of parental behavior for children. These finings support the importance of parental behavior, not just attitudes and rules in children’s weight status (31).

Despite a number of strengths, including a validated survey, a good response rate, and a rigorous interview protocol, a number of limitations should be mentioned. First, the sample size was relatively small and limited power for subgroup comparisons. In addition, height and weight in the study were self-reported by the parent or caregiver, and although self-reported weight has been shown to be reliable in a previous study, with correlations of *r* = 0.99 with actual weight and less than 2% of individuals systematically underreporting their weight (32), it is possible that respondents misreported their child’s height and weight in a manner that would bias the observed results. For example, the study was collected in an urban area and represents families living in the city where obesity might be of concern, rather than rural areas of China where underweight may be more of a concern than obesity. Also the US CDC cutoffs were used to measure obesity. These differ slightly from the WHO cutoffs and may have resulted in less children being classified as obese if different cutoff scores were used.

## Conclusion

The rates of childhood obesity in Nanchang, China were quite high compared to historical trends observed in previous Chinese samples. This finding is consistent with other studies from China, which indicate that the rate of childhood obesity is rapidly rising and may soon surpass developed nations. Our study suggests that family meals times and parents’ use of computers and television may have an important impact on childhood obesity status. However, it seems to be more of a cumulative effect of several variables rather than one or two strong factors. Future studies are needed to explore the roles of the home and neighborhood environment to understand how the changing cultural and environmental dynamics are effecting the development of childhood obesity. Interventions to address parenting practices and improve the home environment in a Chinese context are needed.

## Ethical Approval

The study was approved by the Institutional Review Boards at the University of Hawaii at Manoa and Nanchang University in accordance with the ethical standards of the Helsinki Declaration. Participants were all adults and provided verbal consent to maintain their anonymity. This manuscript is currently unpublished and not under consideration for publication elsewhere.

## Author Contributions

PZ drafted the initial manuscript and conducted the data analysis; HW assisted in the drafting of the manuscript; XZ led the data collection team and critically revised the manuscript for intellectual content; YL, ZY, and JBM critically revised the manuscript for intellectual content; JEM designed the study, conducted some analyses, and critically revised the manuscript for intellectual content. All authors contributed significantly to the interpretation of the data.

## Conflict of Interest Statement

The authors declare that the research was conducted in the absence of any commercial or financial relationships that could be construed as a potential conflict of interest.
